# Generating and leveraging explanations of AI/ML models in materials and manufacturing research

**DOI:** 10.1016/j.patter.2025.101340

**Published:** 2025-08-11

**Authors:** Erick J. Braham, Jennifer M. Ruddock, James O. Hardin

**Affiliations:** 1 Materials and Manufacturing Directorate, Air Force Research Laboratory, 2977 Hobson Way, Wright-Patterson AFB, OH 45433-7126, USA; 2 AV, Inc., 4401 Dayton-Xenia Road, Dayton, OH 45432, USA

## Abstract

In some technical domains, machine learning (ML) tools, typically used with large datasets, must be adapted to small datasets, opaque design spaces, and expensive data generation. Specifically, generating data in many materials or manufacturing contexts can be expensive in time, materials, and expertise. Additionally, the “thought process” of complex “black box” ML models is often obscure to key stakeholders. This limitation can result in inefficient or dangerous predictions when errors in data processing or model training go unnoticed. Methods of generating human-interpretable explanations of complex models, called explainable artificial intelligence (XAI), can provide the insight needed to prevent these problems. In this review, we briefly present XAI methods and outline how XAI can also inform future behavior. These examples illustrate how XAI can improve manufacturing output, physical understanding, and feature engineering. We present guidance on using XAI in materials science and manufacturing research with the aid of demonstrative examples from literature.

## The bigger picture

The rapid rise of predictive models has unlocked transformative opportunities in science and engineering, but it has also introduced serious challenges. Chief among them is the “black box” problem, where complex models make decisions through layers of opaque computations. This lack of transparency has led to real-world errors with grave consequences, including poor personnel decisions, misdiagnosed illnesses, and deceptive chatbots. Such failures have fueled skepticism about the role of artificial intelligence (AI) and machine learning (ML) in making critical decisions. Explainable AI (XAI) is a set of techniques that have been developed in response to these new challenges, offering tools to peer inside these black boxes and revealing how specific features drive predictions. XAI has been shown to strengthen fairness and accountability in human-centered applications. Beyond these origins, XAI holds immense promise for materials science and manufacturing, where costly, low-data environments amplify the need for interpreting models. Here, XAI can illuminate physical mechanisms behind statistical patterns, guide safer and more effective process design, enable models to transfer across related challenges, and ultimately foster confidence in AI-driven innovations that support worker safety, materials discovery, and advanced manufacturing.

## Teaser

Explainable AI (XAI) has emerged to address the “black box” problem in predictive modeling, offering transparency in AI decision making and helping to prevent critical failures in high-stakes environments. This review provides insight into the current state of XAI and the motivations for its uses, with a focus on bridging the gap between technical XAI developments and domain-specific applications in manufacturing and materials science.

## Introduction

### The need for explanations in research

The past two decades have seen machine learning (ML) applied to a broad range of problems from automating mundane tasks to making decisions in life-and-death situations.^1^ Many ML strategies were developed in the context of “big data” such as user data aggregated online, large gnomic datasets, and many more scenarios where the volume of data exceeds human processing capabilities. The data volume in some science and engineering research, however, can be much smaller due to the cost of experimentation, simulation, materials, and more. There are ways to compensate for the lack of data when adapting ML methods to applications in science and engineering, such as using a physics-informed neural network to incorporate physical laws while training a model or using open-source neural networks pre-trained on big datasets to facilitate transfer learning. As an ideal but uncommon example, convolutional neural networks originally developed for image classification were adapted to find patterns in one-dimensional (1D) data streams like disease markers in genomic data or anomaly detection in the vibration of an electric motor.^2^ This progress has been accelerated by community efforts to review and disseminate the use of ML methods into the specific context of scientific/engineering communities, including our own materials and manufacturing community.^3^^,^^4^

State-of-the-art predictive models in research and development have become increasingly complex with the widespread use of methods such as deep convolutional neural networks (CNNs), large language models (LLMs), and generative adversarial networks (GANs). These models can have incredible utility, accuracy, and flexibility but come at the cost of transparency; often appearing as a “black box” where the relationship between inputs and outputs is too complex and opaque for a human to understand. Explainable artificial intelligence (XAI) seeks to tackle this challenge with methods that provide insight into predictive ML models in various contexts. XAI, as with the wave of ML adaptation before it, requires adaptation and dissemination to bridge the knowledge gap from its data-science foundation to the broader range of domain researchers that could benefit from these strategies. Reviews^1^^,^^5^^,^^6^^,^^7^^,^^8^^,^^9^^,^^10^^,^^11^ of the breadth of XAI methods and uses are a great starting point toward understanding the field, but examples in a specific discipline’s context can accelerate the adoption into that discipline more directly.^12^^,^^13^^,^^14^

This review seeks to introduce the core methodologies in XAI via illustrative examples of its use as it has been adapted in materials and manufacturing research. XAI-enabled explanations have the ability to make predictive models easier to navigate, develop, and trust. In materials and manufacturing research, data-driven models have been used in many topic areas, for example, materials design/discovery, process optimization, robotics/automation, 3D printing/additive manufacturing, and defect detection. Possessing an understanding of which feature or input variable in an experiment is driving the output of a model can lead to valuable insights that guide future experimental decisions. XAI explanations, when describing the details of feature relationships, can aid a researcher in uncovering mechanistic insight into the system they are analyzing. For instance, a model using elemental composition to predict a material property such as hardness or crystal structure may be interrogated to find correlated atomistic features that indicate a physical mechanism. The potential insight to be gained is just one aspect of what motivates the use of XAI. Explanations can benefit a variety of concerned parties, including researchers, users, or third parties impacted by the model’s decisions. For instance, workers on a manufacturing floor who use a model’s output to make decisions can better interpret the results and make adjustments to a process without the need for insight into the model’s nature.^15^^,^^16^ The motivations that drive the development and use of XAI methods stem from several origins, all of which may apply to the materials and manufacturing research domain.

### Broader context of XAI

When using opaque models in areas that impact or involve people, such as manufacturing, building a human-interpretable understanding is necessary. These people, public and practitioners, may have different motives behind seeking explanations but may overlap in certain goals and solutions.^6^ In general, public stakeholders focus on the straightforward explanations and identifying risk in the human impact of the model’s decisions, while practitioners focus on understanding and gaining insight into the causes of a model’s behavior. Other publications have addressed this range of motivations. For clarity, we will briefly define core explanation intentions, starting with those more likely to motivate the public, then moving to those more likely to motivate the practitioners. Specifically, we will describe the need for explanation in terms of fairness, informativeness, transferability, trustworthiness, and causality.

Fairness is the XAI goal that involves how these models can impact human safety/wellness and has instigated the drafting of legislation mandating transparency and explanations when using artificial intelligence (AI) or ML models in public settings. Recent initiatives from the European Union^17^^,^^18^ and the United States^19^ to formalize explainability in fields such as defense/justice policy and medical policy seek to ensure fairness when predictive models are used in high-stakes situations by enshrining a “right to an explanation” in addition to more traditional ethical requirements.^20^^,^^21^ Several studies motivated these legal measures by showing that unjustified bias is a common and alarming problem when training on data from improperly curated population data.^22^^,^^23^^,^^24^ These errors can result in functional, ethical, or even legal problems in systems trying to automate hiring, medical diagnosis, and or recidivism likelihood.^23^^,^^25^ Fairness is relevant in manufacturing settings where a variety of humans interact with a model. For example, a natural language model in a process may not fully comprehend certain accents and cause unanticipated failures. Some could inappropriately and illegally attribute these failures to causes other than the model.

Informativeness is the intention of creating a more human-understandable interpretation of a model’s results and process. A large list of coefficients, weights, biases, and rules may be a technical explanation as to why a model is behaving in a certain way but cannot be interpreted by most humans. Creating ways to understand models more holistically is one of the earliest developments of XAI,^6^^,^^26^^,^^27^ but the type of explanation and level of complexity depends on the target audience. Explainability in ML is first and foremost a social process, and what qualifies as sufficiently explainable is context dependent when achieving a goal of informativeness.^20^ While some engineers might be content with the information that a model is behaving as expected, researchers may seek informativeness as a step toward causal/mechanistic insight when interrogating a model.

Transferability in predictive models addresses using a model in contexts beyond their training. Transfer learning is a large field of ML looking into strategies to achieve flexible models that have the ability to work and adapt to multiple circumstances. For instance, a researcher may want to use a model for identifying defects in steel to analyze defects in more costly titanium by transferring the model with some adjustments but limited additional training. The pursuit of transferable models can be aided by explanations and, therefore, XAI tools. XAI analyses focusing on transferability will probe the model for problematic sensitivities, though not necessarily with a specific transfer target application in mind. When a model is transferred to a new dataset, an XAI analysis can be applied to both sides of the transfer situation as a tool to evaluate consistency in the explainable aspects of a problem.

Trustworthiness, as a goal, means building a model that a practitioner can trust will behave consistently when presented with similar inputs. Trust in this context means how much a model is relied on and what the consequences of an error are allowed to be. A model’s trustworthiness is the justification for how much trust to give.^28^ In the simplest case, trustworthiness is often increased by training on a large and complete dataset such that there is minimal error. When training data are limited, as is often the case of materials or manufacturing research, an appropriate calibration of trust can be achieved through model explanations. Even for a trustworthy model, having an understanding of failure modes is important for making correct decisions regardless of the model’s predictions. XAI strategies that aim to show tolerance to perturbation, outlined in the following sections, represent one way of looking into building a more trustworthy model.

Causality is an intention in the vein of informativeness that specifically seeks to find correlations between the input features and predictions of a model as well as precedence and evidence that the correlations are causal. In essence, causality seeks to inform whether there is evidence present that makes a stronger causal case than simple correlation. The requirements to establish causality from observational data in general is a broadly studied topic.^29^ Due to the volume of information needed to confidently make a case for causality, it is a goal that often proves difficult to confidently assert. XAI can provide data-informed evidence for a broader causal theory that includes known physical principles. One way ML models are used in materials research is by using a data-driven surrogate model to predict the output of first-principles calculations to save on computer resources. These models aim to mimic the causal relationship of the physics-based systems and upon interrogation should show behavior congruent with the analogous simulation output.

XAI tools’ initial adoption into materials and manufacturing research has been primarily in the realm of explaining feature importance with the general goals aligning with the intentions outlined above as informativeness and causality.^12^^,^^13^^,^^15^ To give greater context throughout this work, we will highlight which XAI explanation intention each example is addressing. We aim to provide insight into the current state of XAI methods and research and examine the ways in which it has thus far been adapted and used in the context of materials development and manufacturing research.

## Current methods of model and dataset interrogation and their uses in materials research

This section aims to provide an overview of different XAI techniques, along with examples from materials and manufacturing research. It also includes relevant context to newer techniques, such as older or related algorithms. The discussion is organized by how the explanations can be attained: model-agnostic methods, deep-learning-specific methods, and interpretable models. Throughout this section, examples of XAI applications are used to illustrate ways XAI can be adapted to answer technical questions and bring research insights.

### Model-agnostic methods

Explanations that are not dependent on the model type are called model-agnostic methods. When interrogating a model, one can think along two dimensions, as seen in Table 1.^35^ The first dimension is “what to explain?”: whether to examine a single instance of an input-output pairing or to examine the entire dataset. The second dimension is “how to explain?”: whether to interrogate a single feature of the dataset or all the features of the dataset. To illustrate these techniques, we will be using a dataset of multiple principle element alloys (MPEAs).^36^ For each alloy, this dataset contains the atomic fractions of each elemental component, the phases present (face-centered cubic [FCC], body-centered cubic [BCC], hexagonal close packing [HCP], or intermetallic [IM]) and the Vickers hardness, which will be the target variable. Alloys with missing phase data are listed as not available (NA). This is a very simplified version of the dataset that does not consider other material properties, processing methods, or more detailed phase information. Other work has used the full dataset to train ML models for property prediction and generative alloy prediction.^37^^,^^38^ Here we are using it to train a simple model as a materials-relevant example.

**Table 1:** Explanation methods by how and what they explain in a model

What to explain?	How to explain?	
	All features	Single feature
Instance	SHAP attribution (Lundberg and Lee^30^)	individual conditional expectation/ceteris paribus (Goldstein et al.^32^)
	LIME (Ribeiro et al.^31^)/surrogate models	
	salience maps (CNNs)	
Dataset	SHAP importance	SHAP dependence
	permutation importance	partial dependence (Friedman^33^)
	tree-based importance	accumulated local effects (Apley and Zhu^34^)
	surrogate models	

At an instance level, the most commonly used model-agnostic methods for looking at the attributions of all features (the upper left entry of Table 1) are Shapley additive explanations (SHAP) and locally interpretable model-agnostic explanations (LIME).^30^^,^^31^ With SHAP, each feature value of an input instance additively contributes to the output value. “Breakdown attributions” are calculated by going feature by feature and determining the contribution of that feature to the result based on the average prediction for those feature values. However, breakdown attributions depend on the order in which each feature’s attributions are applied.

**Figure 1:**
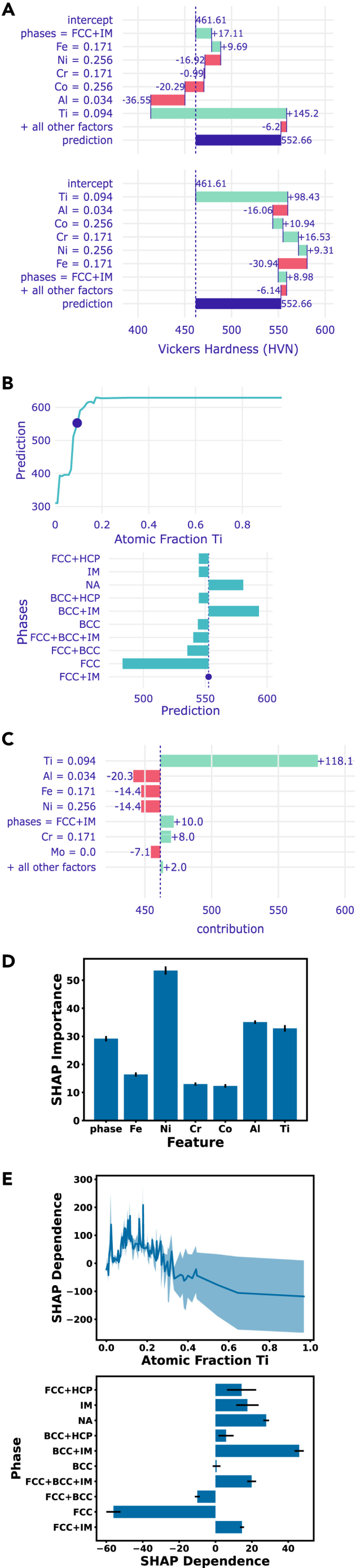
Examples of model-agnostic XAI methods on the MPEA dataset XAI plots for a single input to a model trained on a simplified subset of MPEA dataset, showing the challenges of these methods.^36^ (A) Two breakdown attribution plots for the same input, with the feature attributions applied in reverse order from each other. The mean Vickers hardness for the dataset is 462.5, while the predicted hardness for this input is 552.58. (B) Ceteris paribus plots for the same input as the breakdown plots. Top: the effect of changing the atomic fraction of titanium. Bottom: the effect of having different phases present in the alloy. (C–E) SHAP methods. (C) SHAP attributions for the same input as in (A). (D) SHAP importance as taken from averaging SHAP attributions across the dataset. (E) SHAP dependence for the same features as in (B), as taken by averaging SHAP attributions. Error bars and shaded regions reflect standard deviations.

Breakdown attributions can be seen in Figure 1A for an instance of the MPEA dataset, in which the top plot shows the attributions for one ordering of the features, and the bottom plot shows the attributions for the reverse order of the features. In the top plot the “Fe” feature has a positive attribution, while the opposite is the case for the bottom plot. The average predicted Vickers hardness for any alloy in the dataset is 462.5. The top plot shows that any alloy with both FCC and IM phases (FCC+IM) increases the average hardness prediction by 16.9–479.4, while an atomic percentage of 0.171 iron increases the average hardness of any FCC+IM alloy by 8.3. Thus the contribution of the “Fe” feature is +8.3 in the top plot. However, if the features are reversed the contribution of the “Fe” feature is −34.74, as seen in the bottom plot. SHAP overcomes this ordering issue by iterating over all possible orderings (or a random subset of orderings) of the features and averaging the contributions, as in Figure 1C. Performing SHAP on one instance is called SHAP attribution.

LIME, on the other hand, creates a surrogate model to describe the data space near (local to) a given instance. LIME works by taking an instance and perturbing it and seeing how the perturbations affect prediction outcomes. This can then be used to train an interpretable model that works for that instance, typically a linear regression or a decision tree. A drawback of LIME is that the applied perturbations could result in infeasible “data” used to make the interpretable model—for example, perturbing an alloy to have an energetically unstable phase present. Since SHAP averages breakdown attributions, it may also hide correlated features. These issues of addressing correlated features are common with XAI methods. SHAP and LIME provide different means of probing the way a model treats a given instance and can complement each other in interrogating feature attributions for a given input-output pairing.

Individual conditional expectation (ICE) profiles are a common technique for visualizing the effect of a single feature on a single instance (upper right entry of Table 1). ICE profiles are also called ceteris paribus (CP) profiles, Latin for “all else being equal.”^32^ Examples are shown in Figure 1B for both a continuous feature (atomic fraction of Ti) and a categorical feature (phase) for the same alloy used in the breakdown attribution plots. The top plot shows the effect of only changing the atomic fraction of titanium on the predicted hardness of the instance. The SHAP breakdown plots in Figure 1A show a large positive contribution for the “Ti” feature, consistent with the CP plot. The bottom plot of Figure 1B shows how the predicted hardness changes if different phases are present in the alloy. This correlation aligns with the generally understood trend that the presence of intermetallic phases increases hardness, while FCC tends to decrease hardness. This type of mechanistic affirmation aligns with the XAI intention of causality, whereby the analysis of a model can provide supporting evidence of a physical phenomenon. However, it is important to consider a model’s use of correlated features when investigating causality. Uncovering data-supported evidence for mechanisms is an advantageous use of XAI in materials research.

For some dataset-level methods (lower left entry of Table 1), instance-level methods can be averaged to obtain feature importances. Feature importances measure the magnitude of a feature’s effect on a model’s prediction, unlike the instance-level attributions. In addition to averaged SHAP and LIME feature importances (Figure 1D), there is also permutation importance and occlusion importance. With permutation importance, each of the features of the dataset are individually permuted, and the effect of the permutation on the prediction accuracy determines its importance score. The occlusion method is similar, but instead each feature is “occluded” by setting it to zero or some mean value (e.g., “graying out” image pixels), after which an importance score is determined by the magnitude of the response.

For ensemble-based models, there are additional methods for generating feature importances and surrogate models. The tree-based feature importance is also called Gini importance but can be used with any type of model criterion. This tree-based importance of a feature takes the decision nodes involving that feature and calculates how much the evaluation criterion (Gini or otherwise) is reduced by those nodes. Tree-based importance is often deemed unreliable compared to permutation importance.

In addition to feature importances, surrogate models can describe a model’s behavior globally. There are various algorithms for creating surrogate model explanations, but the only requirement is that the surrogate model is more interpretable than the ML model that needs an explanation. For example, the ElemNet model is trained to take the fractional composition of each of 86 elements and predict material properties based on that composition. Wang et al. trained a decision tree as a surrogate model to predict ElemNet’s formation energy predictions.^39^ The decision tree’s feature importance showed that the more electronegative elements were more important for formation energy prediction. This confirms our known understanding of chemistry. Similarly, Kharal used a simplified list of rules as a surrogate for a random forest model trained to classify steel plate defects.^40^ Another example is the use of a decision-tree surrogate model to interpret a thin-film transistor liquid crystal display defect classification model. Lee et al. found that they could use the decision tree to identify when the ML model was likely to misclassify an image.^41^ While their surrogate model did not confirm known theory, it does allow for a better calibration of how to best use their ML model.

Lastly, we have the lower right entry of Table 1: methods for looking at how an individual feature affects the predictions of an entire dataset. SHAP dependence is simply plotted by taking the average SHAP value for a single feature for each instance in the dataset (Figure 1E). Partial dependence (PD) profiles are the result of calculating the ICE/CP profiles for every instance (or a subset of instances) and averaging them. PD profiles, like CP, do not control for correlations. One might naively approach this problem by looking at local (or marginal effects): Only calculating the CP for an instance using a “local” range of the desired feature. However this has the effect of ignoring (marginalizing over) any correlated features by associating the correlated effect to just the desired feature. Accumulated local effects (ALE) is a method that may not eliminate this issue but will alleviate it.^34^ ALE, rather than taking a simple average, works by averaging across other features and accumulating (summing/integrating) the local effects for the desired feature value range, thus mitigating the effects of other features. While ALE might seem like the only method worth using, it can still be important to look at PD profiles and SHAP to note any differences that should be investigated.

Now that the individual sections of Table 1 and Figure 1 are explained, it is important to note how the different methods can work together to build a bigger picture of the dataset and model. Figures 1B and 1C show trends for a single input, while Figures 1D and 1E show averaged SHAP values for the entire dataset. Comparing Figures 1B and 1E, the phase plots show similar trends, with large positive values for BCC+IM and NA phases, and negative values for the FCC phase. This is to be expected for an input that is “typical” to the dataset. However, the titanium plots show opposite trends for high-atomic-fraction titanium. This may seem contradictory, but it reflects how SHAP dependence and CP are fundamentally different calculations. For SHAP, the average attribution of Ti decreases as its fraction increases. However, for the CP plot, the predicted outcome increases to a plateau as the Ti fraction increases. This apparent conflict can be attributed to the specific input used to calculate the CP plots. That input probably exists in a subset of the data that exhibits different behavior from the majority. To glean a better understanding, more exploratory data analysis such as clustering or histogramming may be necessary. The SHAP plot shows the mean dependence, but the large shaded region suggests that while the mean is negative, the positive CP result may not be uncommon.

Figures 1C and 1D are less easy to directly compare than Figures 1C and 1E. Figure 1D shows a high importance for Ni while Figure 1C shows only a small negative attribution. However, since this is a regressor model, a small negative attribution does not mean that Ni is unimportant to the prediction. It likely means that a nickel fraction of 0.256 is somewhere near the mean and therefore does not shift the prediction far from the mean prediction. However, it might be interesting to explore why this input has such a strong positive attribution for Ti, especially in light of the comparisons of Figures 1B and 1E. Analyzing the different sections of Figure 1 in this way helps elucidate how the different XAI methods complement each other to bring forth a greater understanding of the dataset and how the model is using it to make predictions.

Model-agnostic interrogations can be seen impacting and improving a diverse set of topics in materials research. Sometimes XAI is performed just to see the most important features in determining material properties^42^^,^^43^ or in defect classification.^40^^,^^44^ More interesting case studies can be seen in alloys research, in which Lee et al.^45^ used a combination of SHAP, CP, and breakdown profiles to analyze feature trends and discover that alloy compositions with high mean melting temperatures are predicted to promote BCC phase formation. In both cases the use of XAI is driven by the goal of causality whereby explanations of features help build evidence to support the mechanistic conclusions. Another notable example from materials research is a study by Roy et al., where SHAP was performed on an alloy oxidation model to determine future formulations to try to add to their dataset.^46^ Also, in the field of infrared thermography, Daghigh et al. performed feature importance on their defect detection model to aid feature engineering of their model’s performance.^47^

**Figure 2:**
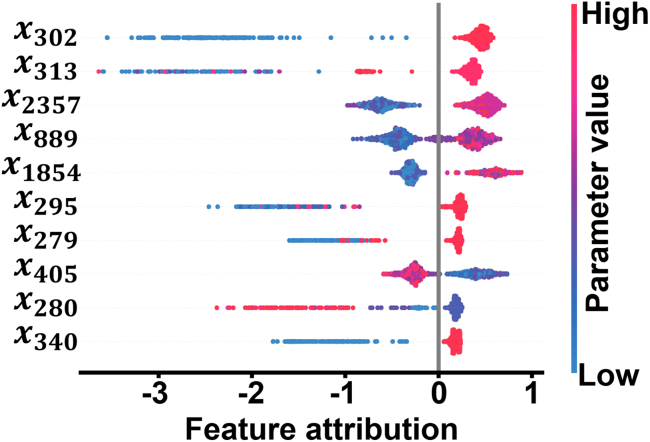
SHAP attributions from a semiconductor manufacturing process model SHAP feature attribution scores for the top ten parameters with the biggest influence on product quality as per the analysis. Parameters, encoded as *x*_*n*_ in the manufacturing process model represent actions possible to be taken in the manufacture of semiconductors. Parameters with high feature-attribution scores were chosen as variables for batch improvement actions in the case study experiment. Adapted, with permission, from Senoner et al.^16^

One other interesting case from the field of manufacturing research is reported by Senoner et al.,^16^ where XAI was used to quantify improvement in semiconductor transistor manufacturing yield. In this approach, a manufacturing system is modeled as a series of sequential manufacturing processes. Each manufacturing process has its own parameters, which may have their own interdependencies. Thus, Senoner et al. came up with their own definition of “process importance,” which was a function of the attribution values for the parameters associated with a given process. Figure 2 shows the SHAP responses for the top ten most impactful parameters, encoded as *x*_*n*_ in the manufacturing process model. These parameters have high correlations between parameter value and feature attribution, as seen by the clustering of red and blue for each parameter. The feature attributions have large magnitudes compared to other parameters. The SHAP values could then also be used to identify which actions to take with those most important processes to improve outcomes. A field experiment confirmed the effectiveness of this model, resulting in an improvement in production yield of 21.7% in trials adjusting the identified parameters. The application of SHAP allowed for a way to rank feature importance and attribution of parameters that may be nonlinear or correlated across processes, two weaknesses that are present in traditional data-driven process optimization. This work is an excellent example of a case driven by the informativeness goal of XAI whereby the explanation allows insight into a complex model that can be more easily interpreted by a human and thus guide future decision making—in this case, finding the most important processes to optimize within a manufacturing system. Another notable example of XAI in manufacturing process analysis was performed by Puthanveettil Madathil et al., where interpretable model design (see “interpretable models” below) coupled with SHAP was used to elucidate the process-product relationship in laser ablation and wire EDM manufacturing case studies.^48^

Table 1 can help guide the breadth and scope desired from an explanation, but there are still many XAI methods to choose from, each with its own limitations. Practicality may prevent using a method like SHAP, which can be computationally expensive compared to permutation feature importance or LIME. Research has gone into evaluating XAI methods to determine their reliability and fidelity to a model.^49^^,^^50^ However, the consensus seems to be to use multiple complementary methods to avoid the pitfalls of one singular method, such as in our interpretation of the different methods used in Figure 1. Using multiple methods allows for cross-verification of the results: if the methods appear to agree, the explanations are likely more robust. Conversely, if the methods do not agree, this might point to correlated features or multimodal distributions that should be further interrogated to better understand the nature of the dataset. Some XAI software packages have built-in evaluation metrics, such as compareXAI and OpenXAI, which can help with choosing XAI methods.^51^^,^^52^

As of this writing, dalex is an excellent available XAI library because of its high-quality application programming interface (API) documentation and breadth of available XAI tools. SHAP, LIME, and CP can all be implemented with dalex^53^; for instance, the plots in Figure 1 were constructed using this package. More specifically, we used the predict_parts method of the Explainer class, as seen in the dalex tutorial (https://ema.drwhy.ai/). Additionally, permutation feature importance can be implemented with the scikit-learn package. There is also interest in making explanations more interactive and conversational, which is seen in the modelStudio package,^35^ an interactive implementation of dalex, and TalkToModel,^54^ a framework that uses LLMs to interactively explain an ML model. For example, someone can ask “how does the presence of niobium affect the hardness of an alloy?” and get a response showing an ICE plot of the hardness of alloys with different amounts of niobium. These interactive methods have an advantage of allowing someone to iteratively ask questions and then get responses that contain only information that is of interest. In this way, model explanations can cater to human curiosity without obfuscating an explanation with excessive detail.

### Deep-learning-specific methods

In addition to the methods mentioned above, there are also those developed specifically for deep neural networks (DNNs). Here, we focus on CNNs because of their long history and relevance to analyzing image-based data in materials and manufacturing.^55^ Imaging is often used for evaluating defects in materials. Many of the methods outlined here can also be applied to other types of models such as natural language models, multi-layer perceptrons, and graph neural networks. These DNN methods often have the advantage of not depending directly on permuting or perturbing variables. However, these methods are not as mathematically established and can yield results that are very subtle or noisy.

**Figure 3:**
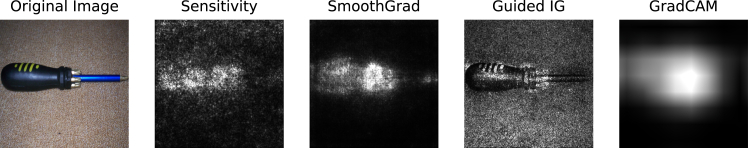
Examples of different types of salience maps Examples of different types of salience maps using an image of a screwdriver on the pre-trained ImageNet InceptionV3 model.^117^ The original sensitivity method is visibly noisy and diffuse, while methods like SmoothGrad, Guided IG, and Grad-CAM can reduce noise. Different methods are often combined, such as combining Guided BackProp with IG to get Guided IG. Grad-CAM is a computationally efficient method but does not have pixel-wise resolution. These salience maps were created using Google PAIR’s Saliency Library.^93^

CNNs have achieved great success in image classification^56^^,^^57^^,^^58^ but also lack interpretability, due to the millions of model parameters they contain. Many techniques have been developed for creating “salience maps”: heatmaps that highlight the regions of an input image that are important for a CNN’s prediction, such as identifying a screwdriver in a manufacturing context (Figure 3) for future use of the tool or identifying potential foreign objects that could damage other processes (walking person or turbine engine). These methods have been grouped and briefly described in Table 2. The importance or relevance of a feature can be interpreted as the gradient of the output with respect to that feature. With that in mind, the first attempts at creating salience maps involved calculating stepwise gradients to back-propagate through the model layers.^56^^,^^59^ These gradient-based approaches, often called “sensitivity” methods, were groundbreaking but often suffered from delocalized or noisy salience maps. This noise is due to the discretized nature of pixelated images and CNN architectures. The discontinuity of ReLu activation functions can also result in unrealistic gradients and model saturation, in which changing an activation value does not affect the output.^6^

**Table 2:** Deep-learning-specific and CNN-specific methods

Method	Brief description	Characteristics
Gradients/sensitivity (Simonyan and Zisserman,^56^ Baehrens et al.,^59^ Zeiler and Fergus^60^)	early methods of back-propagation based on gradients	salience maps can be noisy and disperse
DeConvNet (Springenberg et al.^61^)		
Guided BackProp (Kindermans et al.^62^)		
PatternNet (Smilkov et al.^63^)		
PatternAttribution (Smilkov et al.^63^)		
SmoothGrad (Hooker et al.^64^)		
SmoothGradSquared (Adebayo et al.^65^)		
VarGrad (Shrikumar et al.^66^)		
DeepLIFT (Grad ∗ Input) (Shrikumar et al.,^67^ Sundararajan et al.^68^)	later methods of back-propagation based on gradients, typically by trying to overcome gradient discontinuities	pixel-wise attribution
Integrated gradients (IG) (Erion et al.^69^)		
Expectation gradients (Bach et al.^70^)		
Layerwise relevance propagation (LRP) (Montavon et al.,^71^ Zhou et al.^72^)		
Class activation mapping (CAM) (Selvaraju et al.^73^)	global average pooling final convolutional layer with the weights associated with an output decision to create a salience map	• very easy to calculate • requires very specific CNN architecture • does not give a pixel-wise salience map
Grad-CAM (Bau et al.^74^)	combines CAM with gradient-based methods	inherits characteristics of the combined methods
Guided Grad-CAM (Bau et al.^74^)		
Network dissection (Kim et al.^75^)	find individual CNN units that are associated with pre-defined semantic concepts	to analyze image features, the images need to be semantically segmented and labeled
Testing with concept activation vectors (t-CAV) (LeCun et al.^76^)	find how well a given class or input is associated with a concept	needs examples and counterexamples of the concept in order to train a CAV

Many newer methods attempt to address the disadvantages of simple gradient back-propagation. One way to handle noisy salience maps is smoothing by averaging the salience maps of noisy images using a method such as SmoothGrad.^64^ Another approach adapted to help create sparser, more localized salience maps is by treating the ReLu activation functions and max-pooling layers differently, often filtering out smaller or less-positive activations. The first of this type of method is deconvolutional networks (DeConvNet),^61^ while a popular successor is Guided BackProp.^62^ There are “attributional” methods that try to provide additive pixel-wise salience maps while also addressing the issues of the simple gradient approach. These methods include layerwise relevance propagation (LRP),^71^^,^^77^ integrated gradients (IG),^69^ and deep learning important features (DeepLIFT).^68^^,^^78^ Figure 3 illustrates how these different types of salience methods appear using an ImageNet model. We can see that the sensitivity model highlights the screwdriver but is also somewhat noisy, while SmoothGrad helps remove some of that noise. Guided IG provides a detailed pixel-wise heatmap but also appears to recreate the original image. This is one of the drawbacks of the attributional methods, but it can be mediated through adjustments in implementation.

A different salience-mapping approach, class activation mapping (CAM), uses global average pooling of the final convolutional layer with the weights associated with an output decision to create a salience map.^73^ This method is limited to fully convolutional networks. Grad-CAM overcomes this by taking the final convolutional layer and associating it with a given class through the gradients of the fully connected layers.^74^ Guided Grad-CAM takes this method further by performing an element-wise product with guided back-propagation (BackProp) results.^74^ Often, the choice of salience method can come down to computational cost vs. precision. CAM is fast but of lower resolution than a method like Guided IG (Figure 3).

In the materials domain, salience images are often used in defect detection to confirm that the XAI-highlighted regions of the image are in the locations of the defects.^41^^,^^79^^,^^80^^,^^81^ However, salience metrics have also been used on 1D data to identify patterns in data. When used on a model of battery-cycling data to predict battery lifetimes, salience metrics found significant differences between short- and long-lived batteries.^82^ In an electrospray mode classification model, CAM was performed to find the most relevant frequencies present between the different modes.^83^ In both cases, salience mapping helped with acquiring a better understanding of the data.

**Figure 4:**
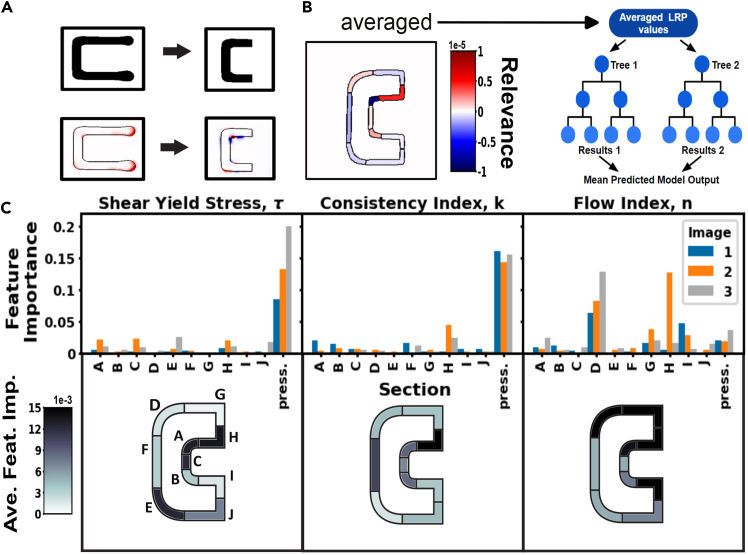
LRP salience maps for feature engineering and semantic analysis in 3D-printed patterns (A) Overhead photos of a simple C-shape pattern were processed to separate the printed filament from the background. Layerwise relevance propagation (LRP) salience maps are shown below the C-shape images before and after halving the C shape. (B) LRP analysis. The segment-averaged LRP salience map is used as random forest input to predict the CNN output. (C) Permutation feature importances on the test set averaged over 1,000 iterations showing importance of image areas and pressures. The C-shape segments are labeled A–J as shown, and the average feature importance for each segment across the three images is shown using a color map. Adapted from Weeks et al.^84^ under CC-BY license.

Deep-model analysis XAI strategies can also help inform materials and process development research. Weeks et al. trained a machine-agnostic *in situ* rheology model to be used in direct-ink-write 3D printing.^84^ They used images of a simple printed C-shape pattern to train a CNN to predict rheological properties of the extruded materials. However, after their initial training, the LRP salience maps revealed that the model was picking up on features that are dominated by printer-specific behavior, as depicted in Figure 4A. Thus, they were able to retrain the model on a dataset of new images that would allow for transferability to patterns printed from a different printer. The salience maps for the C-shape images picked up on the ends of the C shapes, which are dominated by printer-specific pump behavior. To make the data more printer agnostic, the ends were cropped off, yielding more balanced salience maps.

After the new model was trained, Weeks et al. then evaluated the new LRP salience maps by segmenting out different semantic regions of the pattern and averaging the LRP values in those regions to train a simple model, which could then be evaluated for feature importance (Figure 4B). This segmentation and averaging process is an ad hoc dimensionality reduction, allowing for semantic analysis of the salience maps. The results in Figure 4C align with our understanding of the material flow behavior, as the pressure used to extrude is predominantly determined by the yield stress and consistency index, while the flow index determines the degree of shear thinning of the material. These results help with improving trustworthiness by combining the ML results with expert knowledge of the physics while also improving informativeness, since expert knowledge alone would not have been able to determine rheological properties from the images.

**Figure 5:**
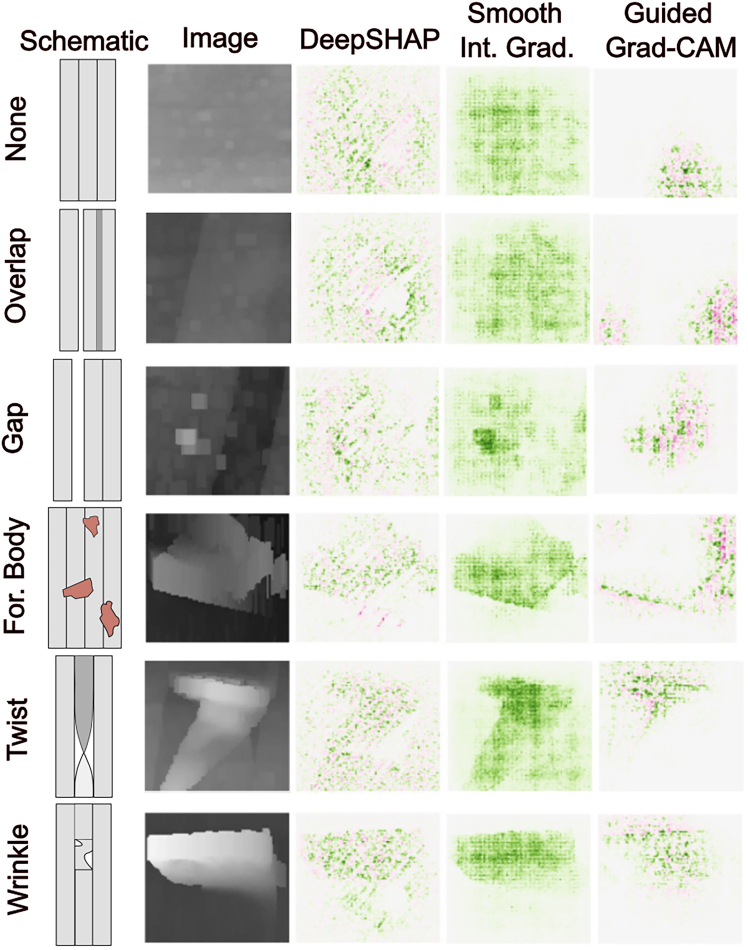
Several types of salience maps for a fiber layup defect classification model Schematics and salience maps of images of different categories of fiber layup defects: no defect (none), overlap, gap, foreign body, twist, and wrinkle defects. Green indicates positive salience, and red indicates negative salience. Adapted from Meister et al.^85^ under CC-BY license.

XAI can help with identifying misclassifications, providing an evaluation of trustworthiness. An example is with fiber layup defect classification, in which Meister et al. trained a CNN and then used Smooth IG, guided gradient CAM (Guided Grad-CAM), and deep learning important features with SHAP (DeepSHAP) to create salience maps (Figure 5).^85^ In their research, they used laser line scan sensor (LLSS) imaging to obtain topological images of deposited carbon fiber reinforced plastic (CFRP) used to manufacture composite parts. The “wrinkles,” “twists,” and “foreign bodies” classes have the most distinctive defects, which show up most apparently with Smooth IG, while DeepSHAP images highlighted those regions but were noisy, and Guided Grad-CAM would only partially align with defects. The “none,” “overlap,” and “gap” defects were less distinctive and resulted in a chessboard-like pattern for Smooth IG, which was attributed to the CNN architecture. DeepSHAP again had noisy patterns for the three classes, while Guided Grad-CAM again had partial alignment with defects. Guided Grad-CAM would also pick out distinct regions of importance for homogeneous “none” images. The authors attributed this to artifacts with some of the pre-processing and smoothing of the LLSS images.

The CFRP salience maps were then evaluated using two metrics: sensitivity and infidelity scores. The sensitivity score calculates the maximum sensitivity of an XAI method to infinitesimally small perturbations to input data. The infidelity metric measures the relevance of individual pixels by “removing” (graying) them. Using these pixel manipulation techniques, they would gradually increase the number of altered pixels, choosing pixels based on how important they were deemed by these salience methods. They found that manipulating <5% of the pixels often resulted in misclassification of images as the “foreign objects” category, regardless of which salience method they used to choose pixels. This is because foreign objects often have different reflective properties than CFRP, and therefore the LLSS images would capture this different signal quality rather than geometry changes, resulting in more image artifacts for that category. These misclassifications often required the fewest number of manipulated pixels when the pixels were chosen using the DeepSHAP importance. DeepSHAP was also seen to have the highest sensitivity score, while all methods had very high infidelity scores with very large standard deviations. This indicated that while DeepSHAP was noisy, it did perform better than the other methods for this dataset.

The researchers proposed that these evaluation metrics could be used for identifying misclassifications or even for identifying an “ambiguous” class. This ambiguous class could be used to identify images that are likely to be misclassified, perhaps because of image artifacts, so that human intervention could be used instead. This is similar in scope to the use of a surrogate model by Lee et al. to address misclassifications, as mentioned earlier.^41^ This type of analysis is helpful for choosing an appropriate calibration of trust to determine situations where more or less human intervention is needed. These analyses can help in choosing classes and can, ironically, improve trustworthiness. Whether the predictions are correct or not, a user can be informed about their correctness.

With all the different salience methods, it can be difficult to choose which ones to use. Legacy methods like the original sensitivity approach and DeConvNet are typically no longer used in favor of newer methods like SmoothGrad, Grad-CAM, IG, LRP, and Guided BackProp. There is research looking specifically at evaluating salience map techniques for CNNs. These methods include checking the sensitivity of these methods to changes in model parameters,^66^^,^^86^^,^^87^^,^^88^ changes to the data,^89^^,^^90^^,^^91^^,^^92^ or changing the data labels.^66^^,^^87^ One major finding of these investigations is that many salience methods were insensitive to randomization of parameters in deeper layers, meaning that if the model weights and biases were randomized in deeper CNN layers, the salience map generated would be largely unaffected. In fact, these methods may just be regenerating the original input image by mostly working as an edge detector and therefore do not rely on the CNN model parameters. This seems especially the case for Guided BackProp. In a similar vein, changing images with a constant shift in RGB values can result in a constant shift in salience maps.^89^^,^^90^ This is especially true for methods that require some type of reference comparison like DeepLIFT, IG, and LRP. Thus, the salience for these methods is not necessarily absolute but relative to the original image. Adebayo et al. showed that adding spurious correlations in a dataset may not be caught by a salience map.^91^ However, many of the metrics around analyzing these salience methods can yield high variance, and without “ground-truth” salience maps it is difficult to choose the best methods.^90^

Other methods for evaluating CNNs are network dissection and testing with concept activation vectors (t-CAV).^75^^,^^76^ These methods attempt to identify specific semantic objects or concepts such as colors or textures that the model is picking up on to make its decisions. Network dissection identifies individual or small numbers of units in a model that are largely responsible for model decisions. In semantically segmented images, certain model units give high activations in the regions of specific semantic objects. If those units are turned “off” (their activations set to 0), the model fails to identify those objects. This helps to show, for example, that a model that can classify airport photos relies on a node for identifying airplanes. t-CAV allows for identifying human-friendly concepts through a concept activation vector (CAV). The CAV is learned by feeding it images that are examples of this concept along with other images and by using the activations of a layer to train a classifier to identify the boundary between the activations associated with the concept and the activations associated with other images. The CAV is the vector that defines this boundary, and it can be used to identify concepts present in an input image. Methods such as network dissection and t-CAV that focus more on identifying semantic concepts can be used in materials research to identify, for example, specific textures or patterns in micrographs, electron backscatter images, or microstructure images.

The best method to use to evaluate a model depends on the type of model and the goals of the user. A salience map may be enough to confirm that a classifier is “looking” at a specific region of interest. Because a straightforward gradient method generally produces noisy salience maps, methods like Grad-CAM, SmoothGrad, and LRP seem to be most popular. Because research often involves regressors or identifying objects and patterns that are less human interpretable, a salience map may require more interpretation through application of segmentation as in Weeks et al., calculating metrics as in Meister et al., or applying occlusions or perturbations. Analysis of semantic concepts via network dissection or t-CAV allows for a more informative interrogation of models but requires more preparation in terms of image segmentation and finding examples of concepts. There are some software packages now available for implementing many of these methods. Google PAIR’s Saliency Library^93^ was specifically developed to work with salience methods (Figure 2) and works with both Tensorflow and PyTorch. For PyTorch, there is captum, which has implementations for most of the salience map methods, along with t-CAV, LIME, and SHAP, and methods for interpreting open-source language models. A package out of Harvard is OpenXAI, which has fewer methods available but also has an evaluator method for evaluating the generated explanation.^52^

In this section we chose to focus on CNNs because of their established use and many associated XAI techniques. However, there are also methods associated with other deep-learning models. For graph models, there is a Harvard package called GraphXAI that appears useful for graph neural network interpretation.^94^ A new challenge in XAI is with LLMs, where there is recent interest in applying them to manufacturing contexts. While there are LLM XAI methods such as attention-based methods,^95^^,^^96^ token importance assignment,^97^^,^^98^ and chain-of-thought reasoning,^99^ and evaluation techniques for these methods,^52^^,^^99^^,^^100^^,^^101^^,^^102^^,^^103^^,^^104^ their application to closed-source models is either impossible or extremely limited. The only way to currently get an explanation from a closed-source LLM is to prompt the model for an explanation,^105^ and evaluating that explanation requires further prompting,^106^ which can be onerous depending on the model API. Ultimately it seems unlikely that an explanation from a closed-source LLM will be anything but dubious. LLM explainability is still very new, and there will hopefully be further research and methods still to come out.

### Interpretable models

Another way to achieve explainability is to use innately interpretable models. These methods may not have the predictive performance of other models, but they have the advantage of avoiding some of the disadvantages of XAI methods. For example, research has shown that XAI methods are vulnerable to adversarial attacks, whereby the model’s output can be minimally affected while the explanation can be drastically affected.^107^^,^^108^ Consequently, innately interpretable models could be especially desirable for high-stakes situations.

When it comes to interpretable models, one often thinks of linear regressions or decision trees. For example, a logistic regression can be used to identify the compounds present in time-of-flight secondary ion mass spectroscopy data.^109^ The weights of the logistic regression model are effectively feature importances. Regularization methods like LASSO^110^ and ElasticNet^111^ trim down the number of features used by a model while maintaining a desired performance metric. Flaschel et al. devised a method of performing ElasticNet on unsupervised constitutive models, resulting in sparser and more interpretable stress fields.^112^ While these methods can yield highly interpretable results, being inherently more informative, they can result in marginalizing overcorrelated variables or leaving out important information.

There has also been research into making DNNs more interpretable. One method is using self-explaining neural networks (SENNs).^113^ SENNs use a unique architecture in which the model has a concept encoder that converts the data into a feature basis of interpretable concepts used by the model. These encoded inputs are then parameterized by their relevance. Interpretable CNNs have optimized filters in the higher layers to pick out specific features associated with a given class.^114^ These methods can in a way output their own semantic concepts using these filters or feature bases similar to t-CAV or network dissection. Multi-explanation graph attention networks are graph models that use an attention network to generate explanations by masking unimportant features.^115^ This method has been used on mutagenicity data and solubility data to identify relevant functional groups and chemical motifs.

Another avenue for model interpretability is to perhaps view some models as partially interpretable. For example, transformer models rely on encoding inputs with an attention mechanism. One can pull out these embeddings and examine how different parts of an input are attending to each other.^95^^,^^96^ Similarly, one can look directly at CNN filters to see what information they are filtering. With recurrent neural networks (RNNs), the parameters of the recurrent cells are reused on time-dependent data. It has been shown that performing principal-component analysis on the recurrent cells in an RNN can pull out evolutionary data or history variables of constitutive models.^116^ This was shown for simulated elastoplastic and viscoplastic data with known models. It would be interesting to see this type of model interpretation on real experimental data.

Both XAI and interpretable models have merits and demerits. Forcing interpretability on a model can result in worse model performance or ignoring important features. However, interpretable models allow users to avoid XAI techniques and look directly at how the model is behaving, making it easier to understand the trustworthiness, which can be useful for particularly high-stakes decisions. Designing interpretable models will probably continue to be an ongoing area of research given the importance of interpretation to users.

## Conclusion: XAI looking forward

The next stages of using ML within the communities of materials and manufacturing research will involve incorporating explanations, whether to gain scientific insight or provide transparency and accountability for an opaque process. Public and nontechnical stakeholders will need the assurance that data-driven decisions are made with fairness and for the correct reasons. Technical stakeholders such as researchers and engineers will benefit from increased usability and informativeness of their models as well as the ability to responsibly build trustworthiness and knowledge when using ML to make discoveries, improve systems, and automate change. Explanations can further the search for causality uncovering the physical meaning behind the features that make formulations successful when designing materials. Engineering is a discipline built on best practices that have been developed over time to provide detailed requirements for safe and trustworthy output. Analogously, responsible use of AI, ML, and XAI is currently developing to provide best practices that ensure safe and trustworthy use of data-driven modeling.

The range of XAI methods and techniques, from model-agnostic approaches to salience maps and concept-based explanations, give us insight into how ML models “think.” Understanding the tools available and the reasoning behind utilizing XAI in materials and manufacturing research is crucial to the successful application of these methods in future work. Modern manufacturing is both rapidly adapting to new technologies and contending with process optimization for high-performance, low-volume, and high-cost components. For example, adapting to new semiconductor materials, such as gallium oxide and silicon carbide, does not have the massive quantities of development data that currently exist for silicon technologies. XAI is a good tool for evaluating the transferability of various approaches that could seek to use silicon data to inform processing on other substrates. XAI as a tool for improving model performance in low-data situations can aid in the development of high-risk, high-reward applications in aerospace (e.g., composites and ceramics) where improved low-data performance is a necessity and improved reliability could benefit many other industries. More specifically, failures in AI-driven composite defect detection should contribute to airframe failures.

As we move forward, it is crucial to recognize that the development and application of XAI methods in materials and manufacturing research are ongoing endeavors. Further research is needed to address challenges such as the interpretability of complex deep-learning models, the development of domain-specific XAI methods, and the integration of XAI into existing materials and manufacturing workflows. The deployment of XAI allows greater insight into the system being modeled and should eventually become a routine step of creating a predictive model. Perhaps tools like model-agnostic explanations will become part of the standard ML workflow bundled together with common software packages. Additionally, AI assistants are now ubiquitous in environments where humans can easily correct defects common in the technology, but such technologies struggle to deal with the high-precision requirements of modern manufacturing automation because of, in part, limitations in the available data. Although XAI tools for transformer technologies, such as LLM chatbots, are in their infancy, XAI-driven insights into such models could lead to more efficient and accurate AI assistants for manufacturing contexts.

## Resource availability

### Lead contact

Requests for further information and resources should be directed to and will be fulfilled by the lead contact, James O. Hardin (james.hardin.11@afrl.af.mil).

### Materials availability

This paper does not generate any new materials.

### Data and code availability

This paper does not report original data or code.

## Acknowledgments

The authors would like to acknowledge very helpful discussions and feedback from Andrew Gillman. This work has been funded by the 10.13039/100006602AFRL.

## Author contributions

E.J.B., conceptualization, writing – original draft, and investigation; J.M.R., conceptualization, writing – original draft, investigation, and visualization; J.O.H., supervision, resources, and writing – review & editing.

## Declaration of interests

The authors declare no competing interests.
